# Expression of Constitutive Nitric Oxide Synthase Isoforms in Varicose Vein Wall; Preliminary Results

**DOI:** 10.1155/2011/204723

**Published:** 2011-05-29

**Authors:** Zora Haviarová, Andrea Janegová, Pavel Janega, Štefan Durdík, Peter Kováč, Viera Štvrtinová, Peter Mráz

**Affiliations:** ^1^Department of Anatomy, Faculty of Medicine, Comenius University, Sasinkova 2-4, 81372 Bratislava, Slovakia; ^2^Department of Pathology, Faculty of Medicine, Comenius University, Sasinkova 2-4, 81372 Bratislava, Slovakia; ^3^Institute of Normal and Pathological Physiology, Slovak Academy of Sciences, Sienkiewiczova 1, 813 71 Bratislava, Slovakia; ^4^Ist Surgical Clinic, University Hospital and Medical Faculty, Comenius University, Mickiewiczova 13, 81369 Bratislava, Slovakia; ^5^Department of Forensic Medicine, Faculty of Medicine, Comenius University, Sasinkova 2-4, 81372 Bratislava, Slovakia; ^6^2nd Internal Clinic, University Hospital and Faculty of Medicine, Comenius University, Mickiewiczova 13, 81369 Bratislava, Slovakia

## Abstract

There are conflicting findings in literature about the structural changes of the primary varicose veins. NO (a potent vasodilatator) is synthesized by nitric oxide synthase (NOS). From 3 known NOS isoforms the two are constitutional: eNOS (endothelial NOS) and nNOS (neuronal NOS). 10 varicose and 10 control vein samples were processed by standard light microscopy and immuno-histochemica techniques using rabbit polyclonal antibodies against eNOS and nNOS. Antibodies expression was evaluated semiquantitatively and proved morphometrically by 2D-image analysis. total area of NOS isoforms expressions was determined by color analysis and color digital subtraction. The results showed discontinuous and significantly lower expression of both NOS isoforms the in the tunica media of varicose veins compared with the control group. For the statistical analysis the unpaired *t*-test was used. Our results suppose lower NO levels in varicose vein wall, deducing that varicose dilatation is due to other mechanism, and they contradict the results of previously published similar works.

## 1. Introduction

Varicose veins are dilated, tortuous, and elongated veins affecting especially the superficial veins of the lower limbs. Beside the post-hrombotic syndrome and crural ulcer, they represent only one of the symptoms of the chronic venous disease—a relatively frequent vascular disease affecting the lower limbs veins of the persons in productive age [1]. The incidence of the chronic venous disease accounts for about 40–60% in females and 15–30% in males in the developed countries of Europe and USA [2]. According to its etiology we distinguish its primary form (cause unknown) and secondary form (occurring usually as a consequence of the survived deep lower limb phlebothrombosis). As the exact cause of the primary form has not yet been revealed, the therapy of this form still resides mainly in reducing the symptoms. The cause of the primary form of varicosis remains to be the subject of interest of several investigators in the world. There is ethiopathogenetic association between

- venous wall weakness associated with alterations in connective tissue and smooth muscle cells [3, 4],
- altered function of the venous endothelium [5],
- venous valve damage [6, 7],
- alterations in microcirculation and venous wall nourishment [8, 9].

### 1.1. Nitric Oxide (NO)

Is an important cellular-signaling molecule, a potent vasodilatator due to the smooth muscle relaxation. It also inhibits platelet adherence and aggregation, reduces adherence of leukocytes to the endothelium [10]. Furthermore, NO has been shown to inhibit DNA synthesis and mitogenesis, and the proliferation of vascular smooth muscle cells. These antiproliferative effects are likely to be mediated by cyclic GMP [11].

### 1.2. Nitric Oxide Synthases (NOS)

from the biochemical point of view, are a family of complex enzymes catalyzing the oxidation of L-arginine to form NO and L-citrulline. The three human NOS isoforms identified to date are: eNOS (endothelial NOS), nNOS (neuronal NOS), and iNOS (inducible NOS). Their genes are found on human chromosomes 7, 12, and 17, respectively, and so they were named for the tissue in which they were first cloned and characterized [10]. vasculoprotective effect of individual NOS isoforms in human organism is not sufficiently clarified yet [12]. Endothelial NOS (eNOS) and neuronal NOS (nNOS) are constitutively expressed, mainly in endothelial cells and nitrergic nerves, respectively, synthesizing a small amount of NO under basal conditions and on stimulation by various agonists. By contrast, inducible NOS (iNOS) is expressed when stimulated by inflammatory stimuli, synthesizing a large amount of NO in a transient manner. The knowledge of nitric oxide synthases (NOSs) is of extreme scientific importance, not only for understanding new pathophysiological mechanisms but also as a target for therapeutic intervention.

### 1.3. Endothelial Constitutive Nitric Oxide Synthase (eNOS)

The role of NO in regulating vascular tone and mediating platelet function is attributable to the ongoing activity of eNOS. It is pharmacologically identical with previously isolated EDRF (endothelium-derived releasing factor), exprimed by the intact endothelium [13]. Inactivation of the eNOS pathway limits the contribution of NO to vessel homeostasis and results in increased vascular tone and platelet adhesion and aggregation. The activity of eNOS is regulated by the intracellular free calcium concentration and calcium- calmodulin complexes. Endothelial NOS is a constitutively expressed protein predominantly associated with the particulate subcellular fraction, suggesting that the native enzyme is a membrane-bound protein. A detailed analysis of the membrane association of eNOS showed that this enzyme is localized to the Golgi apparatus as well as to specific structures in the plasmalemmal membrane called caveolae. The association of eNOS with a region of the plasma membrane in which several key signal-transducing complexes are concentrated (such as G-proteins) is likely to have profound repercussions on enzyme activity as well as on its accessibility to intracellular mechanisms of the pathway release, including mechanisms independent of intracellular calcium release [10].

### 1.4. Neuronal Constitutive Nitric Oxide Synthase (nNOS)

This isoform is present in central and peripheral neuronal cells and certain epithelial cells. Its activity is also regulated by Ca^2+^ and calmodulin. Its functions include long-term regulation of synaptic transmission in the central nervous system, central regulation of blood pressure, smooth muscle relaxation, and vasodilation via peripheral nitrergic nerves. It has also been implicated in neuronal death in cerebrovascular stroke [10]. NO plays also an important role in the pathophysiology of some neurodegenerative diseases. The presence of NO and NOS should be proved indirectly through the histochemic positivity of nicotinamide dinucleotide phosphate diaphorase (NADPHd) [14]. It was proposed that nerve stimulation directly activated the release of NO from nitrergic nerves and, in fact, NO appears to be the dominant neurotransmitter responsible for the nerve-mediated, endothelium-independent vasodilation [13]. Esteban et al. (1998) documented the nNOS presence in the livers of cat and rat; deducing from this that nitrergic fibres should be involved in the blood flow regulation and further metabolic functions of this organ [15].

Abd-El-Aleem et al. (2000) reported increased levels of eNOS and iNOS in the chronic venous ulcers compared with normal skin [16]. Howlader and Smith (2002) found an increased levels of total NO in the blood of patients with severe forms of chronic venous disease (healed venous ulcers and lipodermatosclerosis) [17]. Our study was aimed to find a linkage between the NOSs and the venous dilatation of primary varicose veins. The changes in NOSs isoforms expression should help to elucidate the cause of the vessel wall structural changes of the primary varicosis.

## 2. Material and Methods

10 varicose vein samples of great saphenous veins [18] (5 males and 5 females, see Table 1 for the age structure of varicose vein group of samples) taken by the stripping surgery in years 1997–2005 were compared with 10 control samples of the lower limb superficial veins (7 males and 3 females, see Table 2 for the age structure of the control group of samples) taken from the necroptic material of the Institute of Forensic Medicine (faculty of medicine, Comenius University) in years 1999–2004 with no previous history of any venous disease and no histological signs of venous wall alterations, usually described by varicose vein wall (phlebosclerosis, intimal thickening, fibrosis of tunica media, smooth muscle reduction, and clot formation). The samples were immediately fixed in the buffered formalin, processed by the standard light microscopy method into the paraffin blocks. The sections were then processed by the standard immunohistochemic technique using the rabbit polyclonal antibodies against both constitutive NOS isoforms (eNOS from SantaCruz, USA and nNOS from BioScience, USA). The expression of the antibodies was evaluated semiquantitatively and also proved morphometricaly. By the semi quantitative evaluation, we concentrated on the histologic characteristics of each section and the localization and intensity of marker positivity in the vein wall layers (− negative, +/− irregular positivity, + weak positivity, ++ medium positivity, +++ strong positivity). The semi quantitative analysis was proved morphometricaly by the 2D image analysis (ImageJ 1.34n, National Institute of Health, USA). The total area of NOS constitutive isoform expressions in tunica media was then determined by the color analysis (the brown color of NOS expression) and by the color digital subtraction was determined its portion of the total area of tunica media. For the statistical analysis was used unpaired *t*-test. 

## 3. Results

**Figure 1:**
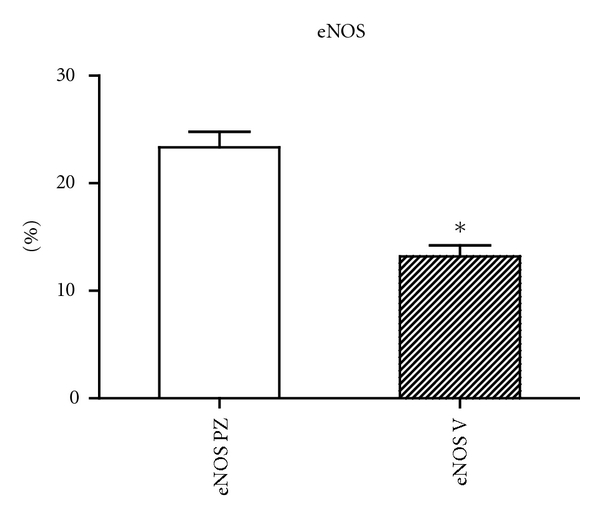
The graph of the morphometric analysis results of the expression of eNOS in varicose and control groups of samples (*P* < .0001).

**Figure 2:**
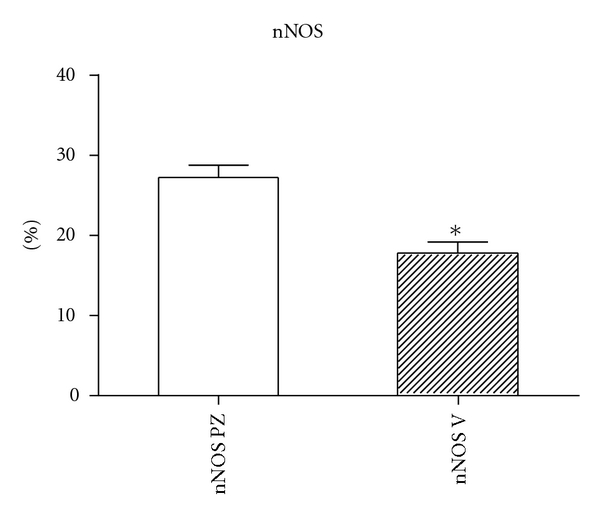
The graph of morphometric analysis results of the expression of nNOS in varicose and control groups of samples (*P* < .0001).

The histomorphological and semi quantitative evaluation of eNOS and nNOS isoforms showed discontinuous and significantly lower expression of both followed constitutive NOS isoforms in the tunica media of the varicose veins compared with control group, where the expression of both NOS isoforms was continuous (see Figures 1 and 2).

**For the Endothelial Isoform of NOS (eNOS)**

**Figure 3:**
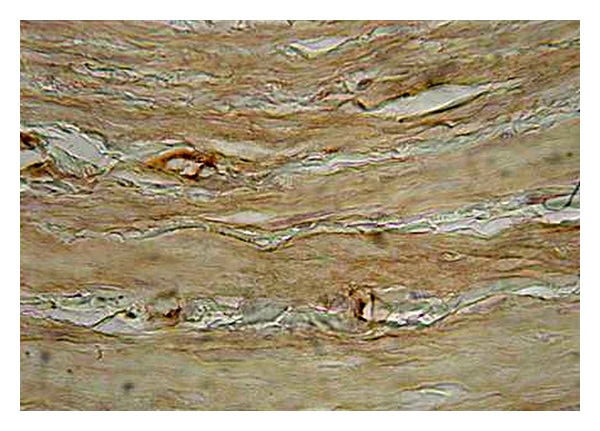
Expression of eNOS in the tunica media of control (healthy) vein (magnification 400x, DAB).

**Figure 4:**
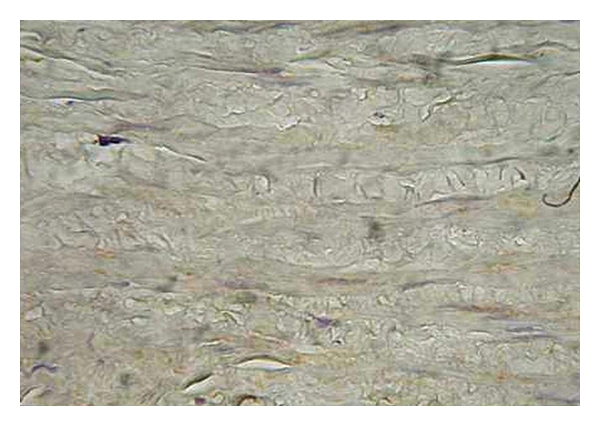
Expression of eNOS in the tunica media of varicose vein (magnification 400x, DAB).

**Figure 5:**
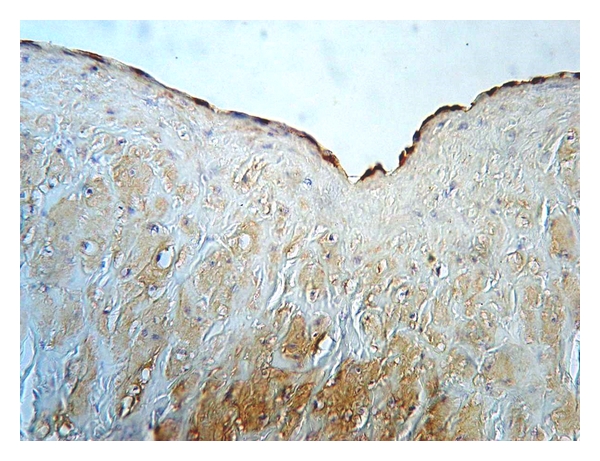
Continuous eNOS cytoplasmatic expression in the endothelial lining of the control (healthy) vein sample (magnification 400x, DAB).

**Figure 6:**
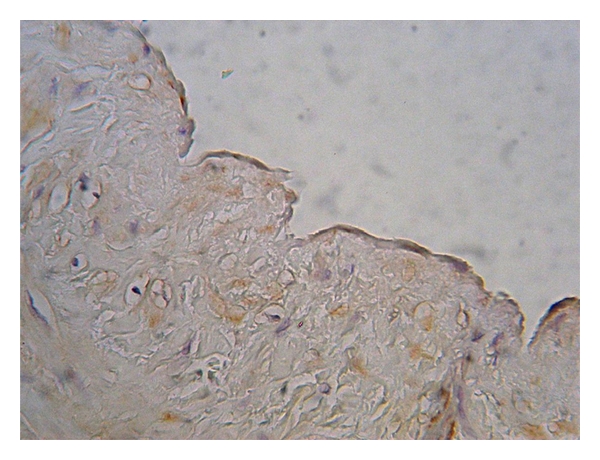
Discontinuous, weak eNOS cytoplasmatic expression in the endothelial lining of the varicose vein sample (magnification 630x, DAB).

We found continuous and diffuse expression of the followed marker in tunica intima and media of the control samples, whereas in the varicose samples, its expression was discontinuous. Enhanced eNOS positivity was observed especially in the endothelial lining of the vessels; in the healthy control samples its expression was continuous over the whole endothelial lining, and in the majority of the varicose samples its positivity showed discontinuity and interruptions in its expression (Figures 3, 4, 5, and 6).

**For the Neuronal NOS Isoform (nNOS)**

**Figure 7:**
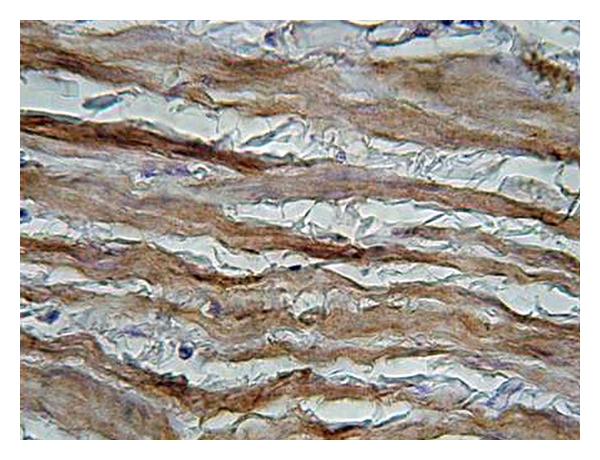
Expression of nNOS in the tunica media of control (healthy) vein sample (magnification 400x, DAB).

**Figure 8:**
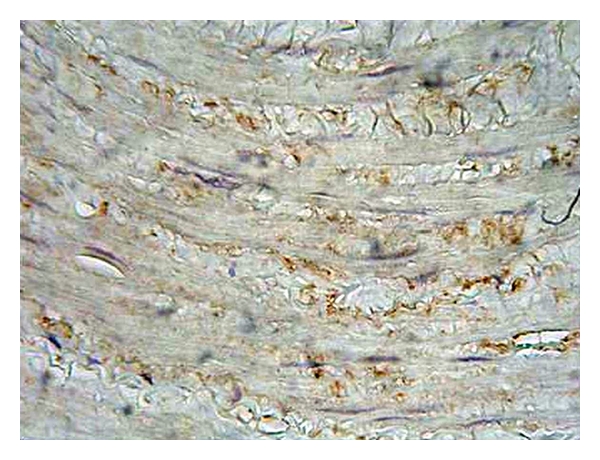
Expression of nNOS in the tunica media of varicose vein sample (magnification 400x, DAB).

**Figure 9:**
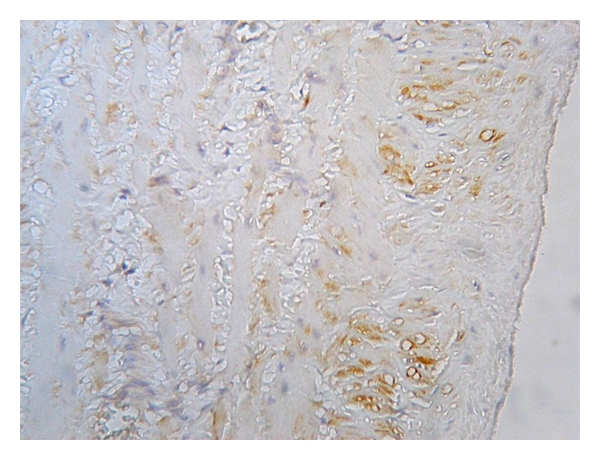
Enhanced nNOS positivity in the tunica intima (compared with tunica media) of varicose vein of 30-yrear-old man (V54), magnification 400x, DAB.

**Figure 10:**
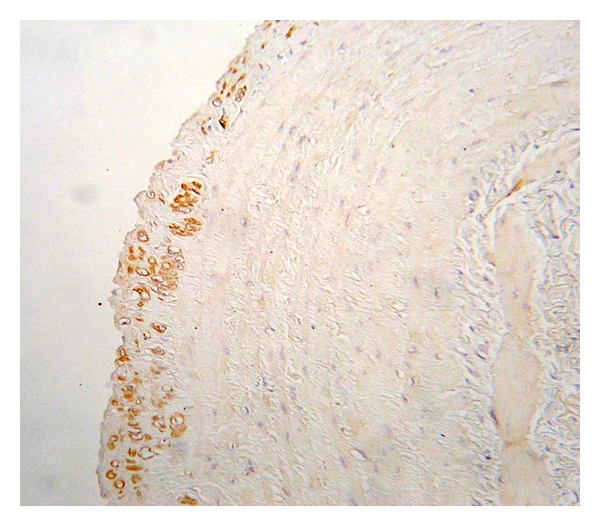
Enhanced nNOS positivity in tunica intima (compared with tunica media) of varicose vein of 32-yrear-old man (V55), magnification 200x, DAB.

The histomorphological and semi quantitative evaluation showed lower and discontinuous expression of nNOS in tunica intima and tunica media of the varicose vein samples in comparison with the control samples (Figures 7 and 8). The only exceptions were two varicose samples (V54, V55); in these varicose samples the nNOS expression was higher in tunica intima than in tunica media (Figures 9 and 10). Both samples represent the varicose great saphenous vein samples of relatively young men (30 and 32 years, resp.), taken by the stripping surgery in the years of 2004 and 2005.

## 4. Discussion

Abd-El-Aleem et al. found increased levels of total NOS, eNOS, iNOS, and arginase in the skin of chronic venous ulcer by using immunocytochemistry, western blotting, and enzyme assays [16]. Howlader and Smith (2002) observed by the colorimetric method raised levels of total NO in the blood plasma of the patients with severe chronic venous disease—healed venous ulceration and lipodermatoclerosiss (corresponding to C4 and C5 of CEAP classification). In the less severe CEAP stages of the chronic venous disease (C2 and C3) the difference of total NO levels between diseased and control subject was not statistically significant [17].

In our study, the stage of CEAP of the varicose (dilated) venous samples was not associated (not marked). So retrospectively, deducing from the fact that these varicose great saphenous veins were decided for extraction (performing the stripping surgery); these venous samples belonged to C2–C6 stages (according to CEAP classification.)

*For the endothelial NOS (eNOS)*, we confirmed the enhanced eNOS expression in the endothelial lining in both followed groups: in the healthy (control) samples, the eNOS expression was diffuse and continuous; whereas in the varicose samples the eNOS expression was discontinuous (Figures 5 and 6). This fact confirms that eNOS is expressed by the intact endothel, and that varicose veins are characterized by impaired endothelial function.

*The expression of nNOS * was also significantly lower and discontinuous in varicose compared with control (healthy) vein samples; deducing that varicose dilatation is due to other mechanisms than potentiated by peripheral nitrergic fibres in the varicose vein wall. The enhanced positivity of nNOS in the tunica intima of two varicose samples (V54 and V55, Figures 9 and 10) is probably due to other unrecognized coincidental disease; by other varicose samples the expression of these two markers in tunica intima copied its expression in tunica media. As was mentioned above, both samples were varicose great saphenous veins of 30 and 32-years-old males (respectively), and there was no difference between the fixation and processing of these two samples compared with the rest of group. 

Our summarized results are contradicting the results of the two above-mentioned studies [16, 17], using two other different methods for the total NOS and eNOS evaluation, which should perhaps be also due to supposed earlier CEAP stages of venous samples in our study (not associated). Nevertheless, our results should be confirmed by the measurement on more numerous sample groups with marked associated CEAP stage of venous disease. From our findings, we can now suppose that enhanced NO is not responsible for varicose dilatation (especially in the earlier CEAP stages), and so the venous wall weakness (the most probable reason of primary varicosis) is a consequence of some other pathophysiological mechanism. 

## 5. Conclusion

Our results of significantly lowered expressions of both constitutive NOS isoforms suppose the lower nitric oxide (NO) levels in varicose vein wall, deducing that the varicose dilatation is due to other mechanism, although the stage of chronic venous disease of the varicose vein samples was undetermined (retrospectively deducing to the C2–C6 stages of CEAP); but we confirmed the impaired function of the varicose vein endothelium. These results shall also suggest that NO level in varicose vein wall should undergo changes depending on the stage of severity of chronic venous disease.

Several possibilities exist for proving the results of our work, we suggest to repeat the method on more numerous groups of samples (with marked CEAP stage of chronic venous disease) to compare Ca2+ presence in vein walls and to check the NOS and NO presence by the indirect histochemic evidence of NADPHd positivity; the comparisons of iNOS expression and the expressions of selected inflammatory stimuli are known for their potentiation of iNOS expression (IL- 1*β*, IL-6, TNF-*α*, etc.) As all NOS isoforms utilize L-arginine as the substrate, with molecular oxygen being the co-substrate [11], L-arginine inhibitors together with ischemia should be considered in the prevention of venous dilatation in case that results of our preliminary work fail to be confirmed by further studies.

## Figures and Tables

**Table 1:** Varicose vein group of samples.

	Males	Females	Together
Number	5	5	10
Average age (in the time of pickup, in years)	29,8	32,8	31,3
Age range (years)	22–37	24–42	22–42
Median (in years)	30	30	30

**Table 2:** Control group of healthy (non-dilated) samples.

	Males	Females	Together
Number	7	3	10
Average age in the time of pickup (in years)	26,1	30,3	27,4
Age range (years)	19–40	20–39	19–40
Median (in years)	26	32	26,5
